# Factors Affecting Utilization of Maternal Health Care Services in Kombolcha District, Eastern Hararghe Zone, Oromia Regional State, Eastern Ethiopia

**DOI:** 10.1155/2014/917058

**Published:** 2014-10-29

**Authors:** Desalew Zelalem Ayele, Bekele Belayihun, Kedir Teji, Desalegn Admassu Ayana

**Affiliations:** ^1^Department of Public Health, College of Health and Medical Sciences, Haramaya University, P.O. Box 235, Harar, Ethiopia; ^2^College of Health and Medical Sciences, School of Nursing, Haramaya University, P.O. Box 235, Harar, Ethiopia; ^3^Department of Medical Laboratory Technology, College of Health and Medical Sciences, Haramaya University, P.O. Box 235, Harar, Ethiopia

## Abstract

*Introduction.* World health organization estimates that more than half a million women lose their lives in the process of reproduction worldwide every year and most of these mortalities are avoidable if mothers have access to maternal health care services. *Objectives.* This study was conducted with objectives of determining the prevalence of utilization of maternal health care services and identifying factors affecting it. *Methodology.* A community based cross-sectional survey was conducted in six kebeles of Kombolcha district. A total of 495 women of reproductive age participated in the study and their selection was made using simple random sampling technique and data was collected using an interviewer-administered structured questionnaire. The data was analyzed using SPSS version 16. *Results.* A total of 495 women were included in this study and from these women about 86.1% had at least one ANC visit during their last pregnancy. About 61.7% of mothers had less than four visits which is less than the recommended and 46.2% started it in the second trimester. Only 25.3% of respondents gave birth in health institutions and rural women were less likely to use institutional delivery 20.9% compared to urban women 35.9%. *Recommendations.* More efforts should be given to educate society in general and mothers in particular, to strengthen community participation and to increase the accessibility of maternal health care services. Moreover, providing accurate information about the services provided in the health institutions is required from the concerned governmental and nongovernmental organizations.

## 1. Introduction

Child bearing is one of the hazardous experiences that women engage in while bringing new life to this world. It is often associated with complications that may cause morbidities, disabilities, and mortalities. World Health Organization (WHO) estimates that more than half a million women lose their lives in the process of reproduction worldwide every year; of these deaths, about 99 percent are from developing countries. The share of Sub-Saharan Africa from the total death toll for developing countries is more than fifty percent and lifetime risk of dying from pregnancy is extremely high; that is, for every 26 mothers, one mother dies as the result of pregnancy and childbirth in Sub-Saharan Africa. This frequency is about 281 times more than the maternal death in more developed countries in which one mother dies from 7300 mothers [1].

In addition to the risk of dying during pregnancy and childbirth, many more women suffer from short and long-term maternal disabilities and illness. According to WHO (2001) for every maternal death, an estimated 30 to 50 women suffer pregnancy related health problems such as vesicovaginal fistulae, infertility, and depression that can be permanently debilitating [2].

Globally more than 70 percent of maternal deaths are due to five major complications (which are direct obstetric complications): hemorrhage (25 percent), infection (15 percent), complication of unsafe abortion (13 percent), hypertension (12 percent), and obstructed labor (8 percent). This complication can occur at any time during pregnancy and childbirth, often without fore warding and often requiring immediate access to emergency obstetric care for their management [3]. The World Bank estimates that 74 percent of maternal deaths could be averted if all women had access to interventions that address complications of pregnancy and childbirth, especially emergency obstetric care [4]. Similarly, studies that focused on maternal morbidity and mortality in developing countries have repeatedly recommended the need for antenatal care and availability of trained personnel to attend women during labor and delivery [5]. ANC provides avenue to provide pregnant women with information, treat existing social and medical conditions, and screen for risk factors. However, it is not enough to receive ANC, since majority of the fatal complications occur during or shortly after delivery. It is therefore important that pregnant women have skilled obstetric attendance during delivery. However, utilization of these services in most developing countries is constrained due to various cultural, socioeconomic, and demographic factors [6]. As the result disparities between developed and developing countries in terms of utilization of antenatal, delivery, and postnatal services are unfairly large, in developed countries, it is estimated that about 97 percent of the pregnant women receive ANC and 99 percent use skilled obstetric service at delivery, whereas in developing countries, only 65 percent and 53 percent of women use ANC and skilled obstetric care services, respectively [7].

Ethiopia is one of the Sub-Saharan African countries that experience the highest maternal mortality ratios in the world; that is, 673 per 100,000 live births and more than fourteen thousand mothers die as the result of pregnancy and related causes each year [8]. In addition, more than 400,000 suffer long-term disabilities due to complications during pregnancy, delivery, or postpartum periods. The use of ANC, delivery, and postnatal services by Ethiopian women is one of the lowest in the world. Almost all births take place at home in Ethiopia (94 percent) with only six percent of women delivering in heath institutions. The majority of the births are assisted by relatives or some other untrained person. Attendance of prenatal care is very low; only 28 percent of all Ethiopian mothers received prenatal care from a trained heath professional. The quality and frequency of this care are variable; many women receive the care either too late or too few times in their pregnancy [8].

Most maternal mortalities and disabilities in the country are due to direct obstetric complications, which are avoidable if women can get adequate and timely antenatal, delivery, and postdelivery services. The major causes of maternal death are abortion (32 percent), obstructed labor (22 percent), hypertension (9 percent), sepsis (12 percent), haemorrhage (10 percent), and others (15 percent) [9]. Reducing maternal morbidities and mortalities is the issue that is given due attention by Ethiopia as the Ethiopian government is among the first African governments to make a strong commitment to the United Nations inspired millennium development goals (MDGs) by making one of the MDG targets, maternal health, central to its national development strategy, that is, reducing maternal mortality by three quarters between 1990 and 2015, which would mean reducing to 250/100,000 live births by 2015 from the 1990 estimate of 990 [10, 11]. However, in spite of the concerted efforts of the Ethiopian government, the level of maternal mortality is not reduced as desired. The 2011EDHS, for instance, estimates the maternal mortality to be 676 maternal deaths per 100,000 live births [12]. Similarly, utilization of maternal health care services is not improved to the extent wanted. Regional variations in terms of the level of utilizing maternal health care services are very high. The uses of these services in Oromia National Regional State are relatively low compared to other regions in the country and the factors for this low utilization in the region in general and for the various parts of this region are not well examined.

The situation of maternal health care utilization in Oromia National Regional State in which the current research is done is low even compared to the national level. The 2005EDHS shows that the proportion of mothers who received ANC, delivered at health institutions, delivered with assistance of health professionals, and made postnatal checkup was 24.8, 4.3, 4.8, and 4.5 percent, respectively, for Oromia National Regional State, while it was 27.6, 5.3, 6.0, and 5.5 percent, respectively, for the country as a whole [8]. Moreover, utilization of maternal health care services was lower for Oromia National Regional State compared to other regions in the country. For example, the use of both delivery and postnatal care services was lower for Oromia Region compared to Addis Ababa, Gambella, Harari, and Dire Dawa regions. The use of antenatal care was higher for urban women (69 percent) than their counterparts in rural areas (23.7 percent) [8]. In general the variations in utilizing maternal health care services and factors affecting its utilization varied by geographic area and socioeconomic and cultural settings in the country which calls for investigation of area and culture specific determinants of maternal health care utilization. However, the magnitude of utilization of maternal health care services and factors affecting it are not investigated in Eastern Hararghe Zone in general and Kombolcha district in particular while this part of the country has its own peculiar geographic, socioeconomic, and cultural setting which might affect utilization of these services. Studies trying to explore factors affecting utilization of maternal health care services in Eastern Hararghe Zone in general and Kombolcha district in particular are very scanty and it is this knowledge gap that leads us to initiate this study. Thus, identifying factors specific to each area so as to identify area specific measures that can help minimize the hindrances is very crucial. The purpose of this research is therefore to identify the major factors that influence the utilization of maternal health care services in Kombolcha district, Eastern Hararghe Zone, Oromia Regional State.

Studies have been done to explore the determinants of maternal health care services utilization in the different parts of the country and these studies have shown that the magnitude of maternal health care services utilization varied by geographic areas and socioeconomic and cultural settings, and similarly the factors determining the utilization of these services varied by geographic area and socioeconomic and cultural settings. Eastern Hararghe Zone of Oromia Region in general and Kombolcha district in particular is the area where the factors that determine utilization of maternal health care services are not assessed and the level of utilization of these services is not examined. Thus, identifying factors peculiar to a given geographic area and socioeconomic and cultural settings is crucial for understanding community level determinants of maternal health care service utilization.

The potential beneficiaries of the results of this study are the society in general and women and children in particular as the interventions made on maternal health care services improve the utilization of these services. Moreover, governmental and nongovernmental organizations working on maternal and child health care may use the results of this study as an input in their planning for improving maternal and child health. The general objective of this study was to assess the factors that affect utilization of maternal health care services and to suggest relevant recommendations that enable the concerned bodies to design relevant intervention strategies in Kombolcha district. The study was conducted in June 2012.

## 2. Methodology

A community based cross-sectional study design that employed quantitative data collection method was carried out to assess factors affecting utilization of maternal health care services in June 2012 in Kombolcha district, Eastern Hararghe Zone, Eastern Ethiopia. Kombolcha district is one of the 19 districts in Eastern Hararghe Zone. Its capital city, MelkaRafu, is located at a distance of 18 kms from Harar town to the north direction. The district had 20 kebeles of which three were urban and 17 were rural [13]. In 2007/2008, the total population of the district was 104,248 of which about 51% were males, while 49% were females. In 2007/2008, there were 13 clinics, 11 health posts, one health center, and one drug shop. The health coverage of the district was about 86% [13].

The study populations were women of reproductive age (15–49 years) who gave at least one live birth in the five years prior to the survey date and who were the usual residents of the district. The sample size was determined using the formula of Cochran *n* = *Z* ^2^ *PQ*/*d* ^2^ [14]. Thus, taking the prevalence of one of the major parameters in this study, that is, antenatal care utilization, which was 24.8 percent (0.25) for Oromia Regional State [8], the sample size was determined. The estimate of the sample in this study was desired to be precise at confidence level of 95 percent and margin of error of four percent (*d*). Then, using the formula *n* = *Z* ^2^ *PQ*/*d* ^2^, the sample was determined to be (1.96)^2^(0.25) × (0.75)/(0.04)^2^ = 450. Adding 10 percent allowance for nonresponse, the total sample size was determined to be 495. To identify the study units, the district was first stratified into urban and rural areas and one urban and five rural kebeles were selected from the district using simple random sampling technique. Then lists of eligible women who live in the selected kebeles were obtained from kebele health extension workers registration books. A probability sample proportional to the population size technique was used to determine the number of respondents that were selected from each kebele. Finally, the respondents included in the study from each kebele were identified by using simple random sampling technique.

The questionnaire was adapted from Ethiopian Demographic and Health Survey English version. It was further developed after reviewing of relevant literatures that address the objectives of the study. After extensive revision, the final version of the English questionnaire was developed and translated to the local language, that is, Afaan Oromo, to make the communication between the data collectors and respondents easy. Pretesting of the questionnaire was made in rural kebele adjacent to the study area, that is, Haramaya kebele, which has similar sociodemographic setting with the study population. Based on the feedback obtained from pretesting further refinement of the questionnaire was made. Ten data collectors who completed minimum of grade ten and can speak local language and two nurses with diploma for supervision were recruited, trained, and assigned as data collectors and supervisors, respectively. In collecting the data, a face-to-face interview technique was used.

The outcome variables in this study are antenatal and delivery care services utilization for which the categories are either use or nonuse of the services. The independent variables were categorized into demographic which include age of women and their husbands, marital status, and place of residence and socioeconomic which includes education of women and their husbands, occupation of women and their husbands, religion of women and their husbands, women's knowledge/attitude towards maternal health care services, availability, and accessibility of maternal health care services, women's perception of quality of services, and cost of transportation and services. The data was entered using EPI INFO version 3.51 and finally was exported to SPSS version 16.0 for analysis. Frequencies and summary statistics such as means, standard deviations, percentages, and ranges were computed to describe the study population in relation to relevant variables. The association and significance between the dependent and independent variables were measured using binary logistic regression analysis.

The ethical clearance was obtained from Haramaya University, College of Health Sciences Research Ethics Review Committee. The survey was commenced after obtaining permission from Eastern Hararghe Zonal Health Department and District Council. Informed verbal consent was obtained from each study subject. Each respondent was informed about the objective of the study and assurance of confidentiality.

## 3. Results

A total of 495 women were included in this study to investigate the factors that influence utilization of maternal health care services. All of the respondents were Muslims in religion, more than 95 percent were married, and more than 60 percent were housewives. The majority of respondents were illiterate (71.9%) and primary level (grades 1 to 8) educated constituted 26.3%. Secondary and above level educated constituted less than two percent. The majority of respondents were in the age category from 20 to 34 which is the peak reproductive age category and about 93.3 percent of the respondents lied in this age category. About 69.9 percent of the respondents had family members attending formal school. 98.2 percent of respondents ever attended health education. About 12.1 percent of respondents ever experienced abortion/still birth. More than 95 percent of the respondents have access to health facility in their own kebele while only 1.8 percent of respondents have no access to health facility in their own kebeles. About 86.1% of respondents attended antenatal care (ANC) for their recent birth. Of these 35.5% started attending ANC in the first trimester, 46.2% in the second trimester, and the remaining 18.3% in the third trimester. Out of 426 mothers who received ANC for their recent pregnancy, 263 (61.7%) made less than four visits while 163 (38.3%) made four and more ANC visits during the course of their recent pregnancy. Only 25.3% of respondents gave birth in health institutions for their recent birth with the help of health professionals while majority (74.7%) of respondents gave birth at home without the help of health professionals. Rural women were less likely to use institutional delivery, 20.9%, as compared to urban women, 35.9%.

Those women who delivered at home were asked why they preferred home and their reasons were as follows: easy labor 281 (75.9%), feeling shame to go to health institutions 42 (11.4%), health facility being far 36 (9.7%), and other reasons 11 (3.0%). Of those who gave birth at home, 255 (69.1%) were attended by TTBA's followed by neighbors 103 (27.9%) and the remaining 12 (3.0%) by others. Those women who delivered at health institution were asked why they prefer health institution and their reasons were as follows: being sick 32 (25.6%), received health education 37 (29.6%), saving mother's life 31 (24.8%), good service 15 (12.0%), no fee 6 (4.8%), and other reasons 4 (3.2%). Of those who gave birth at health institutions 52 (41.3%) were attended by midwifes, followed by nurses 34 (27.0%), medical doctors 17 (13.5%), health extension workers 4 (3.2%), health officers 2 (1.6%), and do not remember 16 (13.5). About 92.1% of husbands of respondents lied in the age category from 25 to 44 years. The major occupation of husbands was farming (90.1%) followed by merchant (7.3%) and the remaining occupations constitute less than three percent. The majority of husbands were illiterate (51.7%) and primary level (grades 1 to 8) educated constituted 44.6%. Secondary and above level educated constituted less than four percent.

The bivariate analysis of factors affecting attendance of ANC indicated that age of women, education of women, health education on maternity of women, presence of health facility in the kebele, family size of the household, history of abortion/still birth, means of transport to the nearest health facility, perception of women to the quality of maternal services, and rural-urban residence were found to be significant predictors of ANC utilization. On the other hand, the bivariate analysis of factors affecting delivery at health institution indicated that occupation of women, education of women, occupation of husband, education of husband, family member/s education, household family size, history of difficult labor, means of transport to the nearest health facility, women's perception of quality of maternal services, and rural-urban residence to be significant predictors of utilization of institutional delivery.

To examine the net effect of the variables on ANC and institutional delivery utilization by controlling for the confounders, the binary logistic regression was fitted. Two models were fitted, that is, one for ANC utilization and one for institutional delivery. As the result, the variables such as age and education of mothers and perception of mothers to quality of maternal services provided were found to be significant predictors of ANC utilization (see Table 1 for detail).

The results of logistic regression for institutional delivery on the other hand showed that variables like occupation of mothers and their husbands, education of husbands, history of difficult labor, and so forth were found to be significant predictors of institutional delivery (see Table 2 for detail).

## 4. Discussion

A total of 495 women were included in the study. 86.1% had at least one ANC visit. 61.7% of mothers had less than four visits and 46.2% of mothers started attending ANC in the second trimester. The finding differs from EDHS 2011 in which 34% of mothers received ANC from skilled providers for their recent births [12]. This could be because EDHS covered more remote areas. But this finding is consistent with the findings of the study done by Zeine et al. in Hadiya Zone in 2009 in which 86.3% of women made at least one ANC visit during the course of their recent pregnancy [15].

About 38.3% of mothers made four and more ANC visits which is higher than the national level in 2011 in which only 19% of mothers made four and more ANC visits [12]. Demographic and socioeconomic variables such as age of women, education of women and their husbands, receiving health education on maternity, presence of health facility in the kebele, presence of family member/s attending formal school, family size, history of abortion/still birth, history of difficult labor, means of transport, perception of quality of maternal services, and rural-urban residence were found to be strongly related to maternal health care services utilization.

In this study, education of women and their husbands remained strong predictor of maternal health care services utilization and these results are consistent with the findings elsewhere [16–18]. Women engaged in farming and merchant activities were more likely to use institutional delivery compared to housewives. Similarly, women married to merchants and others were more likely to use institutional delivery compared to women married to farmers. Mothers who previously experienced obstructed labor were found to be higher users of maternal health care services. This result is consistent with other studies [19]. Similarly, mothers who previously experienced abortion/still birth were better users of maternal health care services than mothers who did not. This could be because of the fact that mothers who had history of obstructed labor/abortion/still birth have practical experience about the dangers associated with pregnancy and childbirth than those who did not and this could motivate them to receive ANC and give birth at health facility.

## 5. Recommendations

Those women with low level of education, women married to husbands with low level of education, women who are unemployed and married to farmers, women from household with no family member attending formal school, women who never experienced difficult labor, abortion/still birth, women who use foot as means of transport, and women who perceived the quality services provided to be low quality and residing in rural areas were greatly disadvantaged in utilizing maternal health care services. Thus, improving education of the population in general and women and girls in particular and availing appropriate package of maternal services to the disadvantaged groups could be an appropriate strategy to utilization of maternal health care services in the area.

Lack of awareness about the dangers associated with pregnancy and childbirth and the feeling that labor is easy at home and feeling shame to go to health institutions for delivery are some of the factors that affect maternal health care services utilization in the study area. Thus, concerted efforts should be made by the concerned stakeholders to teach the negative effects of these attitudes through mobilization activities.

Perception of mothers to the quality of services provided is the other factor influencing it. This could be because of absence of adequate information about the services provided in the health institutions and hence provision of information should be strengthened by the concerned governmental and nongovernmental organizations so as to encourage mothers to utilize maternal health care services.

## Figures and Tables

**Table 1:** The result of logistic regression: the effect of demographic and socioeconomic factors on the use of ANC services.

Characteristics	*B*	Exp(*B*)	Significance	95% CI for Exp(*B*)	
				Lower	Upper
Age of respondent					
15–24^*^	0.373	1.452	0.669	0.262	8.033
25–39	1.694	5.440	0.034	1.133	26.113
40–49 (RC)		1			
Educ. respondent					
Illiterate (RC)		1			
Primary and above	0.951	2.587	0.031	1.090	6.140
Health educ. on maternal health					
Yes^*^	1.158	3.184	0.234	0.472	21.471
No (RC)		1			
Family size					
Below five children (RC)		1			
Five and above five children^*^	0.414	1.514	0.263	0.732	3.128
History of abortion/still birth					
Yes	2.605	13.536	0.000	5.673	32.297
No (RC)		1			
Means of transport to health institution					
Walk (RC)					
Vehicle^*^	1.000	2.718	0.070	0.922	8.013
Perception of quality of services					
Good/very good	2.316	10.136	0.000	2.776	37.006
Bad/very bad (RC)		1			
Rural-urban residence					
Rural (RC)		1			
Urban	1.698	5.463	0.001	1.995	14.957

RC: refers to reference category. ^*^These variables are insignificant at *P* ≤ 0.05.

**Table 2:** The result of logistic regression: the effect of demographic and socioeconomic factors on the use of health institutions for delivery.

Characteristics	*B*	Exp(*B*)	Significance	95% CI for Exp(*B*)	
				Lower	Upper
Occup. of respondent					
Farmer^*^	−0.419	0.658	0.187	0.353	1.255
Merchant	1.428	4.171	0.001	1.762	9.874
House wife (RC)		1			
Educ. respondent					
Illiterate (RC)		1			
Primary and above^*^	0.023	1.024	0.939	0.562	1.865
Occup. husband					
Farmer (RC)		1			
Merchant	1.418	4.128	0.001	1.722	9.896
Others^*^	1.038	2.824	0.126	0.748	10.658
Educ. husband					
Illiterate (RC)		1			
Primary and above	0.533	1.704	0.038	1.031	2.819
Family member attending formal school					
Yes	0.646	1.909	0.012	1.151	3.165
No (RC)		1			
Family size					
Below five children (RC)		1			
Five and above five children^*^	0.218	1.244	0.452	0.704	2.198
History of difficult labor					
Yes	1.398	4.045	0.000	2.290	7.147
No (RC)		1			
Means of transport to health facility					
Walk (RC)		1			
Vehicle	0.972	2.644	0.028	1.109	6.305
Perception of quality of services					
Good/very good	1.216	3.374	0.000	2.076	5.483
Bad/very bad (RC)		1			
Rural-urban residence					
Rural (RC)		1			
Urban^*^	0.802	2.230	0.063	0.957	5.198

RC: refers to reference category. ^*^These variables are insignificant at *P* ≤ 0.05.
